# Co-Detection of ADV, Influenza B, and HPIV: Independent Risk Factors for SMPP with Changes in NPIs

**DOI:** 10.3390/v17091266

**Published:** 2025-09-19

**Authors:** Linlin Huang, Ting Shi

**Affiliations:** 1 Pediatric Intensive Care Unit, Children’s Hospital of Soochow University, Suzhou 215000, China; 15950058982@139.com; 2 Department of Infectious Diseases, Children’s Hospital of Soochow University, Suzhou 215000, China

**Keywords:** severe mycoplasma pneumoniae, children, risk, viral co-detection

## Abstract

**Background:** This study investigated the epidemiology of Mycoplasma pneumoniae (MP) in children with acute respiratory tract infections (ARTIs) and explored the risk factors for severe mycoplasma pneumoniae pneumonia (SMPP) in children. **Methods:** A retrospective analysis was conducted on 36,380 children with acute respiratory infections who underwent multiplex real-time polymerase chain reaction (RT-PCR) assays for nine respiratory pathogens from September 2021 to November 2024. **Results:** A total of 36,380 children with ARTIs were enrolled in this study. The co-detection rate of MP with other pathogens was significantly higher in the post-NPIs period than in the NPIs period (36.5% vs. 25.7%, *p* < 0.01). Multivariate regression identified the detection of influenza A virus (InfA), InfB, human parainfluenza virus (HPIV), human bocaparvovirus (HBoV), human rhinovirus (HRV), adenovirus (ADV), human respiratory syncytial virus (HRSV), and human metapneumovirus (HMPV) as protective factors against MP epidemics (*p* < 0.01); meanwhile, older age, the cancellation of NPIs, and summer–autumn seasons were found to be risk factors. After adjusting for sex, age, period, season, and pathogens, InfB (OR: 3.009, 95%CI: 1.041–8.697, *p* = 0.042), HPIV (OR: 2.226, 95%CI: 1.170–4.235, *p* = 0.015), and ADV (OR: 2.035, 95%CI: 1.105–3.750, *p* = 0.023) were identified as independent risk factors for SMPP. **Conclusions:** These findings highlight post-NPI shifts in MP epidemiology and identify ADV, InfB, and HPIV as early warning markers for SMPP.

## 1. Introduction

Mycoplasma pneumoniae (MP), the smallest prokaryotic pathogenic microorganism, is a major cause of both upper and lower respiratory tract infections [1]. It was found to be responsible for approximately 10–40% of community-acquired pneumonia (CAP) cases in children [2,3]. During an epidemic period, MP was found to be responsible for 30–50% of CAP cases, with up to 18% of affected patients requiring hospitalization [4]. Additionally, MP pneumonia (MPP) occurred more frequently in school-aged children than in adults [3]. Based on the clinical severity of the disease, MPP can be classified into general MPP and severe MPP (SMPP) [5]. SMPP may potentially progress into a life-threatening illness [6], imposing substantial economic and social burden on families and healthcare systems.

In MP epidemics, MP is primarily transmitted via respiratory droplets and aerosols during close person-to-person contact. Epidemic cycles of three to five years have been observed in multiple countries [7]. In China, MP epidemics can occur throughout the year, with incidence being higher in late autumn or winter [3]. Previous studies have also shown a positive correlation between the detection rate of MP and age among children with CAP [1]. Moreover, mixed infection was associated with greater disease severity than single-pathogen infection, and coinfections with viruses and MP have been reported in approximately 27% of cases [8]. During the coronavirus disease 2019 (COVID-19) pandemic, widespread implementation of non-pharmaceutical interventions (NPIs) not only limited the spread of SARS-CoV-2 but also reduced MP transmission [9]. However, after the relaxation of these NPIs, many countries experienced concurrent resurgences of influenza viruses and MP [10]. These changes altered the epidemiological trends of respiratory pathogens and increased the proportion of mixed respiratory pathogen infections [11]. At present, there are conflicting results regarding whether coinfection with respiratory viruses and NPIs influence the epidemiology of MP and exacerbate the severity of MP infections.

Monitoring the epidemiological characteristics of MP and prediction of severe MPP at an early stage are crucial for effective management. As is widely recognized, the prevalence of MP is influenced by multiple factors, such as age, season, mixed infections, and NPIs. To clarify these relationships, this study retrospectively analyzed the large clinical sample dataset for children with acute respiratory tract infections (ARTIs) to identify risk factors for MP epidemics, aiding in the prediction of severe SMPP.

## 2. Materials and Methods

### 2.1. Participants

In this study, hospitalized children with acute respiratory tract infections (ARTIs) were identified through the electronic database of the hospital information system at the Children’s Hospital of Soochow University (SCH) from September 2021 to November 2024. Among them, children who underwent sputum respiratory pathogen assays were included. Exclusion criteria were as follows: aged <1 month or >16 years; presence of congenital immune or metabolic disorders; presence of chronic diseases; prior use of immunosuppressive agents; receipt of blood products within the past 1 month (see flowchart in Supplementary Materials, Figure S1). Data on sex, age, time of pathogen detection, discharge diagnosis, and results of real-time polymerase chain reaction (RT-PCR) for 9 respiratory pathogens were retrieved from the electronic medical system. A total of 36,380 patients (19,881 males and 16,499 females) were included with an average age of (1.65 ± 1.13) years. Patients were divided into four age groups: ≤1 year (Group I), 1–≤3 years (Group II), 3–≤6 years (Group III), and >6 years (Group IV). Based on the geographical and climatic characteristics of China, seasonal classification criteria were defined as follows: March–May were defined as spring; June–August were defined as summer; September–November were defined as autumn; and December–February of the following year were defined as winter.

This retrospective study was approved by the Ethics Committee of Children’s Hospital of Soochow University (No. 2024CS058-30 December 2024). This is retrospective data, the informed consent was waived.

### 2.2. Specimen Collection

The decision to conduct sputum pathogen testing was made by physicians of attending level or above based on the clinical judgment of the child’s condition. Nasopharyngeal secretions were obtained from children with ARTIs within 24 h of hospital admission. Samples were stored at 2–8 °C and transported for testing within 30 min. Sputum specimen collection was performed by two skilled resident physicians.

### 2.3. Detection of Nine Respiratory Pathogens by RT-PCR Capillary Electrophoresis Fragment Analysis

The 9 respiratory pathogens detected included mycoplasma (MP), human parainfluenza virus (HPIV), human bocaparvovirus (HBoV), influenza A virus (InfA), influenza B virus (InfB), human rhinovirus (HRV), human respiratory syncytial virus (HRSV), human metapneumovirus (HMPV), and adenovirus (ADV).

Nasopharyngeal aspirates were thoroughly mixed, and the supernatant was collected. A 2 μL aliquot of the RT-PCR internal reference was added to the positive control, negative control, and each specimen, and then mixed with the DNA extract (Ningbo Haishi Gene Technology Co., Ltd., Ningbo, China). Subsequently, 5 μL of this mixture was combined with 15 μL of the PCR reaction mixture and centrifuged at 2000 rpm for 10 s. The PCR mixture contained primers for 9 respiratory pathogens (Ningbo Haishi Gene Technology Co., Ltd., Ningbo, China). The real-time PCR was performed on a LightCycler 480II instrument (Roche, Basel, Switzerland). The amplification procedure was performed according to the manufacturer’s instructions. Finally, the fluorescence signal intensity was measured by capillary electrophoresis.

### 2.4. Definition of MPP and Severe MPP

The diagnostic criteria for mycoplasma pneumoniae pneumonia (MPP) was based on guideline for diagnosis and treatment of community-acquired pneumonia in children (2019 version) [12]: (1) fever, cough, or abnormal lung auscultation; (2) pulmonary infiltrates identified on chest radiography reported by two certified radiologists; (3) detection of Mycoplasma pneumoniae DNA in lower respiratory tract specimens by RT-PCR. Severe MPP (SMPP) was defined as the presence of one or more of the following manifestations in children with MPP [13]: (1) radiography—infiltration involving two-thirds of one lung, multilobar infiltration, pneumothorax, pleural effusion, lung necrosis, atelectasis, or lung abscess; (2) hypoxemia—cyanosis, markedly increased respiratory rate, chest wall depression, tracheal tugging, nasal flaring, SpO2 < 92%; (3) extrapulmonary complications; (4) high fever (>39 °C) for more than 5 days or fever for more than 7 days without a declining trend in peak temperature; (5) feeding difficulties—reluctance or inability to feed.

### 2.5. Statistical Analysis

The continuous variables and categorical variables were presented as mean ± standard deviation and percentages (%), respectively. They were analyzed by an independent-sample *t*-test or a chi-squared test. Logistic regression analysis was performed to calculate odds ratios (ORs) with 95% confidence intervals (CIs). All statistical analyses were conducted by using IBM SPSS Statistics, version 25.0 (IBM Corp., Armonk, NY, USA). Graphs were generated with GraphPad Prism, version 9 (GraphPad Software, San Diego, CA, USA). *p* < 0.05 was considered statistically significant.

## 3. Results

### 3.1. Demographic Characteristics in Children with ARTIs

A total of 36,380 children with ARTIs were enrolled, including 9554 during the NPIs period (from September 2021 to December 2022) and 26,826 during the post-NPIs period (from January 2023 to November 2024). The monthly average number of inpatients was 597 ± 286 during the NPIs period and 1120 ± 420 during the post-NPIs period, with the difference being statistically significant (*p* < 0.01). During the NPIs period, the proportion of male children with ARTIs was higher than in the post-NPIs period (Table 1). Compared with the NPIs period, the proportion of children with ARTIs aged ≤1 year (34.4% vs. 17.5%, *p* < 0.01) and 1–≤3 years (29.1% vs. 18.4%, *p* < 0.01) decreased in the post-NPIs period. In contrast, the proportions of children aged 3–6 years (23.5% vs. 27.5%, *p* < 0.01) and >6 years (12.4% vs. 36.6%, *p* < 0.01) increased significantly in the post-NPIs period. Notably, the positivity rate of MP among children with ARTIs was significantly higher in the post-NPIs period compared with the NPIs period (33.3% vs. 9.2%, *p* < 0.01).

### 3.2. Epidemiological Trend of MP in Children with ARTIs

**Figure 1:**
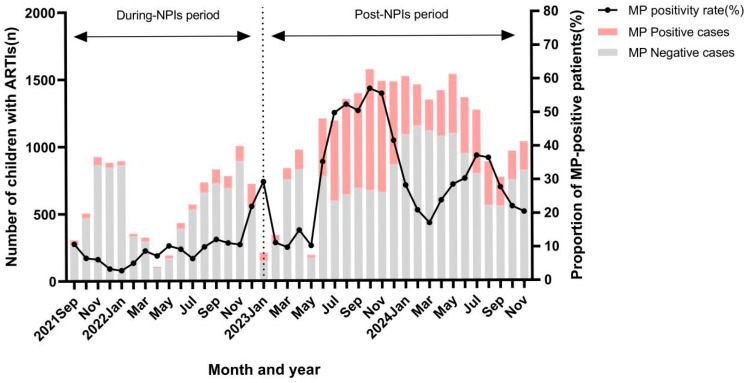
The positive rate and number of MP-PCR tests in children with ARTIs during NPIs and in the post-NPIs period (from September 2021 to November 2024).

As shown in Figure 1, the MP-positive rate remained at a low level during the NPIs period but exhibited two peaks in the post-NPIs period. The first major peak occurred between July and November 2023, and the second smaller peak appeared between July and August 2024. Among MP-positive children, no statistically significant difference was observed in the proportion of male patients between the two periods (53.3% vs. 51.3%, *p* = 0.260, Table 2). Compared with the NPIs period, the proportion of MP-positive children aged ≤1 year (8.0% vs. 4.2%, *p* < 0.01) and 1–≤3 years (17.7% vs. 10.4%, *p* < 0.01) decreased in the post-NPIs period. In contrast, the proportion of children aged >6 years increased significantly (44.5% vs. 58.9%, *p* < 0.01), whereas no statistically significant difference was observed in the 3–≤6 years group (26.8% vs. 26.5%, *p* = 0.846).

### 3.3. Co-Detection of MP with Other Respiratory Pathogens

**Figure 2:**
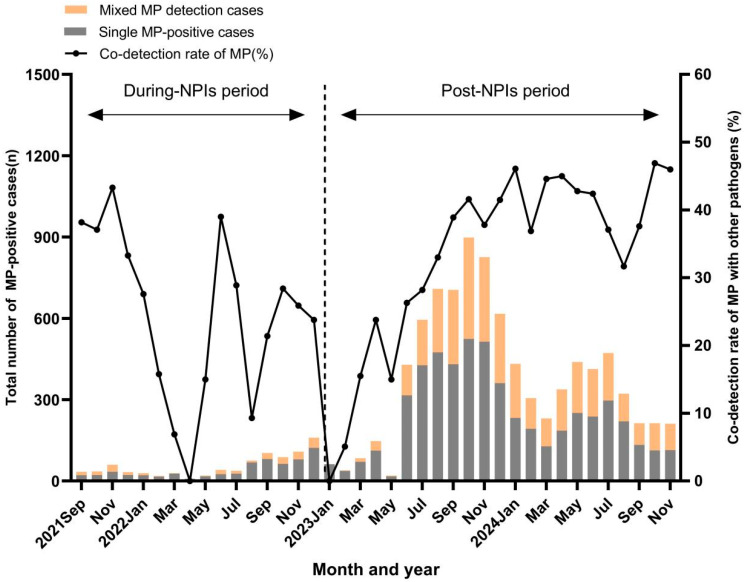
The number and detection rate of other pathogens in patients with ARTIs who had a positive result for MP in the during- and post-NPIs periods (from September 2021 to November 2024).

To explore the relationship between MP and the other pathogens, the epidemic trends of each pathogen are described in Figure S2. During MP epidemic period, HRV, HRSV, ADV, HPIV, and infB also showed high-level prevalence. Compared with the NPIs period, the co-detection rate of MP with other pathogens significantly increased in the post-NPIs period (25.7% vs. 36.5%, *p* < 0.01). During the NPIs period, the most common co-detected pathogen was HRV, followed by HPIV and HMPV (Table 3). After the relaxation of NPIs, the most common co-detections were HRV, ADV, and HPIV. In addition, the co-detection rates of MP with other pathogens fluctuated during the NPIs period; in the post-NPIs period, it showed a gradual increase and subsequently stabilized at a higher level (Figure 2). During the NPIs period, the positivity rates of InfA (1.5% vs. 3.8%; *p* < 0.01), InfB (0.2% vs. 3.3%; *p* < 0.01), HPIV (2.4% vs. 11.6%; *p* < 0.01), HBoV (2.2% vs. 6.5%; *p* < 0.01), HRV (18.2% vs. 24.5%; *p* < 0.01), ADV (0.3% vs. 2.2%; *p* < 0.01), HRSV (2.0% vs. 18.3%; *p* < 0.01), and HMPV (2.3% vs. 12.1%; *p* < 0.01) were each lower in MP-positive group than those in the MP negative group, and the differences were all statistically significant (Table 3). Similar patterns were observed in the post-NPIs period.

### 3.4. Risk Factors for MP Epidemic and Severe MP Pneumonia

As shown in Table 4, two regression models were built to analyze the risk factors for MP epidemic in children with ARTIs. In Model 1 (unadjusted), male, infA, infB, HPIV, HBoV, HRV, ADV, HRSV, and HMPV were identified as protective factors against MP epidemic in children (*p* < 0.01). In contrast, older age, the relaxation of NPIs, and summer and autumn seasons were risk factors for MP epidemics. In Model 2, after adjusting for sex, age, period, season, and co-detected pathogens, sex was found to be no longer statistically significant (*p* = 0.986), whereas all other variables remained significant (*p* < 0.01). As shown in Table 5, in Model 1 (unadjusted), age > 6 years (OR: 0.490, 95% CI: 0.250–0.961, *p* = 0.038), InfB (OR: 3.641, 95% CI: 1.316–10.074, *p* = 0.013), HPIV (OR: 2.113, 95% CI: 1.127–3.962, *p* = 0.020), and ADV (OR: 1.978, 95% CI: 1.081–3.620, *p* = 0.027) were identified as significant risk factors for severe MP pneumonia. After adjusting for sex, age, period, season, and co-detected pathogens in Model 2, only InfB (OR: 3.009, 95% CI: 1.041–8.697, *p* = 0.042), HPIV (OR: 2.226, 95% CI: 1.170–4.235, *p* = 0.015), and ADV (OR: 2.035, 95% CI: 1.105–3.750, *p* = 0.023) remained statistically significant.

## 4. Discussion

In this study, it was observed that the number of children with ARTIs remained at a relatively low level during the NPIs period but increased rapidly after NPIs were lifted. This trend was consistent with the findings of previous studies [14]. Similarly, a delayed but significant rebound of MP epidemic in children was noted after the discontinuation of NPIs. Although the precise cause of the global resurgence of MP in late 2023 remains unclear, several countries have reported that it may be linked to the natural epidemiological cycles of MP [15]. However, other research indicated that the resurgence of MP was driven by a build-up in population susceptibility along with relaxed NPIs, i.e., the so-called “immunity gap” [16]. After strict NPIs were discontinued, ARTIs were more common in children over the age of three rather than in infants or toddlers. One plausible explanation was that children over three years old returned to school, thereby increasing their exposure to respiratory pathogens. In contrast, during the COVID-19 pandemic, younger children (<3 years) demonstrated relatively poor compliance with the NPIs, which may have influenced their infection risk differently [17].

In this study, school-aged children exhibited the highest detection rate of MP both during and after the NPIs periods than other groups, with a higher proportion observed in the post-NPIs period. This finding was consistent with those of previous studies [8,15,18]. This may be explained by the fact that the closed and densely populated nature of schools is particularly conducive to the transmission of MP. Although an MP epidemic can occur throughout a given year, distinct seasonality was observed that was closely linked to ambient temperature. During the NPIs period, the MP positivity rate remained at a low level, and seasonality was not evident. In contrast, following the relaxation of NPIs, seasonality became more pronounced, with a peak occurring from July to September. These findings are consistent with those of the previously reported epidemic patterns of MP in the Suzhou region [19], suggesting that, while the relaxation of NPIs increased the detection rate of MP, it did not alter its seasonal epidemic pattern.

In this study, the co-detection rate of MP with other pathogens was 25.7% during the NPIs period, which was lower than that reported before the COVID-19 pandemic (27.1%) [3]. However, after the relaxation of NPIs, the co-detection rate of MP with other pathogens increased to 32%, exceeding the rates reported in other regions of China [20]. During MP epidemic period, HRV, HRSV, ADV, HPIV, and infB also showed high-level prevalence. Their co-detection rates with MP were 22.0%, 3.5%, 6.2%, 5.1%, and 1.1%, respectively. Rhinovirus (RV) was consistently the most frequently co-detected virus with MP, which may be explained by its ability to colonize the respiratory tract and its high prevalence in recent years [21]. Interestingly, adenovirus (ADV) emerged as the second most common coinfected virus, which may be related to the delayed minor peak of ADV that was observed after the relaxation of NPIs in China [17,22]. Furthermore, it was found that the detection rates of eight respiratory viruses were all lower in MP-positive children than in MP-negative children, which was consistent with the findings of a previous study [3]. This phenomenon may reflect pathogen–pathogen interference, where the presence of one virus reduces the likelihood of coinfection with another [23].

In this study, the multivariate regression analysis indicated that MP epidemics are most common in school-aged children, and that the likelihood of infection increased with age, and was more prevalent in the summer and autumn seasons; these findings are consistent with those of previous studies [8,18,19]. Additionally, the implementation of NPIs and the presence of other pathogens were identified as protective factors against MP epidemics in children, which may be explained by the interruption of MP transmission routes and pathogen–pathogen interference. In recent studies, conflicting results have been derived in investigations into whether coinfection with respiratory viruses was a worsening factor for mycoplasma infections. Some research has indicated that there is no difference in the clinical characteristics, complications, and outcomes between the patients infected with either MP alone or with virus coinfection [24,25,26]. A few factors might contribute to viral colonization, the widespread use of molecular biology techniques, and the timely treatment of the disease [25]. However, several previous studies have indicated that viral coinfections of MP pneumonia frequently led to severe clinical consequences [27,28]. The authors of these studies believe that these viral infections occur primarily in young children, whose respiratory and immune systems are immature and may be more susceptible to other respiratory pathogens, meaning they can develop severe illnesses.

In this study, after adjusting for factors such as sex, age, season, and NPIs, ADV, infB, and HPIV were identified as independent risk factors for SMPP. Previous studies have also revealed that ADA–MP coinfection was associated with severe community-acquired pneumonia in children [6,29,30]. However, to date, little evidence has been found to show that InfB and HPIV exacerbate MP infection severity. Regarding the mechanism by which respiratory viral infections exacerbate MP pneumonia, it has been found that coinfection with viruses and MP can lead to a decrease in the expression of genes related to ciliary activity, alter multiple immune-related signaling cascades and impaired pulmonary defense mechanisms in animal models [31,32].

It is noteworthy that, in this study, the multivariate regression analysis showed that the relaxation of NPIs was not a risk factor for the severity of MP pneumonia. Xu Li et al. proposed that the resurgence of MP in children post-NPIs could be partly attributed to macrolide-resistant MP clones, but this did not impact the primary clinical outcomes [33]. However, some studies have suggested that long COVID-19 leads to the sustained elevation of T-cells and inflammatory factors in the body, which in turn may induce SMPP [16]. Additionally, the severity of MP infection metrics can include other factors (i.e., hospitalization duration, white blood cell count, CRP levels, LDH levels, and the frequency of steroid treatment). But data on these were not available, so were not included here.

Our study has limitations. Firstly, although this study is based on a large sample size, it only explores the risk factors for SMPP from an epidemiological perspective. Secondly, the fact that the data are derived from only one hospital center may reduce the applicability of the results to wider geographical contexts. Lastly, the absence of macrolide-resistance-associated mutation analysis in this study could potentially introduce bias into the results. In future research, we will collect data covering the length of hospitalization, treatment details, and laboratory data to evaluate the risk factors for SMPP from a clinical perspective.

## 5. Conclusions

Compared with the NPIs period, both the positivity rate of MP infection and the co-detection rate of MP with other pathogens increased significantly after NPIs were lifted. We identified that school-aged children are the group who are at a higher risk of MP epidemics during the summer–autumn seasons, whereas the implementation of NPIs and the presence of other respiratory viruses appeared to be protective factors. Importantly, the co-detection of ADV, InfB, and HPIV was found to be an independent risk factor for SMPP.

## Figures and Tables

**Table 1:** Demographic characteristics of patients with ARTIs in Suzhou, China, 2021–2024.

Category	During-NPIs Period (*n* = 9554)	Post-NPIs Period (*n* = 26,826)	*p*
Number of patients with ARTIs (per month)	597 ± 286	1120 ± 420	<0.01
Sex (male, n%)	5394 (56.5)	14,487 (54.0)	<0.01
Age (years, n%)			
≤1	3290 (34.4)	4714 (17.5)	<0.01
1–≤3	2835 (29.7)	4925 (18.4)	<0.01
3–≤6	2246 (23.5)	7382 (27.5)	<0.01
>6	1183 (12.4)	9805 (36.6)	<0.01
Positive rate of MP	880 (9.2)	8942 (33.3)	<0.01

Notes: The data are presented as mean ± SD and n (%). The *t*-test and chi-square test for continuous and categorical variables, respectively. *p* < 0.05 as statistically significant.

**Table 2:** Demographic characteristics of children with ARTIs who were positive for MP.

Category	During-NPIs Period (*n* = 880)	Post-NPIs Period (*n* = 8942)	*p*
Sex (male, n%)	469 (53.3)	4588 (51.3)	0.260
Age (years, n%)			
≤1	70 (8.0)	375 (4.2)	<0.01
1–≤3	156 (17.7)	928 (10.4)	<0.01
3–≤6	236 (26.8)	2371 (26.5)	0.846
>6	391 (44.5)	5268 (58.9)	<0.01
Detection rate of other pathogens	226 (25.7)	3268 (36.5)	<0.01

Notes: The data are presented as n (%). The chi-square test was conducted for the categorical variables. *p* < 0.05 as statistically significant.

**Table 3:** The other pathogens co-detected in MP-positive and MP-negative children with ARTIs.

Pathogens	During-NPIs Period			Post-NPIs Period		
	MP Positive Group (*n* = 880)	MP Negative Group (*n* = 8674)	*p*	MP Positive Group (*n* = 8942)	MP Negative Group (*n* = 17,884)	*p*
InfA (n,%)	13 (1.5)	329 (3.8)	<0.01	221 (2.5)	1195 (6.7)	<0.01
InfB (n,%)	2 (0.2)	288 (3.3)	<0.01	98 (1.1)	528 (3.0)	<0.01
HPIV (n,%)	21 (2.4)	1005 (11.6)	<0.01	457 (5.1)	1703 (9.5)	<0.01
HBoV (n,%)	19 (2.2)	564 (6.5)	<0.01	89 (1.0)	419 (2.3)	<0.01
HRV (n,%)	160 (18.2)	2123 (24.5)	<0.01	1969 (22.0)	4976 (27.8)	<0.01
ADV (n,%)	3 (0.3)	194 (2.2)	<0.01	554 (6.2)	1935 (10.8)	<0.01
HRSV (n,%)	18 (2.0)	1591 (18.3)	<0.01	311 (3.5)	3242 (18.1)	<0.01
HMPV (n,%)	20 (2.3)	1052 (12.1)	<0.01	143 (1.6)	803 (4.5)	<0.01
*p*	<0.01	<0.01		<0.01	<0.01	

Notes: The data are presented as n (%). The chi-square test was conducted for categorical variables. *p* < 0.05 as statistically significant. Abbreviations: InfA, influenza A; InfB, influenza B; HPIV, human parainfluenza virus; HRV, human rhinovirus; ADV, adenovirus; HRSV, human syncytial virus; HMPV, human metapneumovirus.

**Table 4:** Multivariate regression analysis of risk factors for MP epidemic in children with ARTIs.

Variable	Model 1		Model 2	
	OR (95%CI)	*p*	OR (95%CI)	*p*
Sex				
Female	1 (reference)		1 (reference)	
Male	0.840 (0.802–0.880)	<0.01	0.986 (0.934–1.040)	0.986
Age (year)				
≤1	1 (reference)		1 (reference)	
1–≤3	2.758 (2.458–3.095)	<0.01	2.752 (2.443–3.101)	<0.01
3–≤6	6.397 (5.756–7.110)	<0.01	5.140 (4.603–5.739)	<0.01
>6	18.038 (16.278–19.989)	<0.01	10.795 (9.690–12.026)	<0.01
Period				
During-NPIs	1 (reference)		1 (reference)	
Post-NPIs	4.928 (4.578–5.306)	<0.01	3.697 (3.408–4.011)	<0.01
Season				
Spring	1 (reference)		1 (reference)	
Summer	2.197 (2.047–2.357)	<0.01	2.135 (1.971–2.313)	<0.01
Autumn	1.815 (1.695–1.943)	<0.01	2.228 (2.060–2.410)	<0.01
Winter	1.154 (1.068–1.247)	<0.01	1.567 (1.432–1.714)	<0.01
Pathogen				
InfA				
Negative	1 (reference)		1 (reference)	
Positive	0.401 (0.349–0.461)	<0.01	0.232 (0.200–0.270)	<0.01
InfB				
Negative	1 (reference)		1 (reference)	
Positive	0.324 (0.263–0.400)	<0.01	0.220 (0.176–0.276)	<0.01
HPIV				
Negative	1 (reference)		1 (reference)	
Positive	0.451 (0.408–0.498)	<0.01	0.437 (0.392–0.489)	<0.01
HBoV				
Negative	1 (reference)		1 (reference)	
Positive	0.289 (0.237–0.353)	<0.01	0.466 (0.376–0.578)	<0.01
HRV				
Negative	1 (reference)		1 (reference)	
Positive	0.759 (0.718–0.802)	<0.01	0.590 (0.554–0.629)	<0.01
ADV				
Negative	1 (reference)		1 (reference)	
Positive	0.690 (0.626–0.760)	<0.01	0.358 (0.322–0.397)	<0.01
HRSV				
Negative	1 (reference)		1 (reference)	
Positive	0.156 (0.139–0.175)	<0.01	0.250 (0.221–0.282)	<0.01
HMPV				
Negative	1 (reference)		1 (reference)	
Positive	0.225 (0.191–0.264)	<0.01	0.262 (0.220–0.311)	<0.01

Abbreviations: InfA, influenza A; InfB, influenza B; HPIV, human parainfluenza virus; HBoV, human bocaparvovirus; HRV, human rhinovirus; ADV, adenovirus; HRSV, human syncytial virus; HMPV, human metapneumovirus; OR, odds ratio; CI, confidence interval. Model 1 was not adjusted. Model 2 was adjusted for age, sex, period, season, and pathogens.

**Table 5:** Multivariate regression analysis of risk factors for severe MP pneumonia.

Variable	Model 1		Model 2	
	OR (95%CI)	*p*	OR (95%CI)	*p*
Sex				
Female	1 (reference)			
Male	1.166 (0.804–1.690)	0.417		
Age (year)				
≤1	1 (reference)		1 (reference)	
1–≤3	0.446 (0.188–1.058)	0.067	0474 (0.199–1.132)	0.093
3–≤6	0.501 (0.243–1.033)	0.061	0.542 (0.258–1.138)	0.106
>6	0.490 (0.250–0.961)	0.038	0.514 (0.255–1.037)	0.063
Period				
During-NPIs	1 (reference)			
Post-NPIs	0.921 (0.492–1.721)	0.795		
Season				
Spring	1 (reference)			
Summer	0.703 (0.390–1.269)	0.242		
Autumn	0.972 (0.564–1.677)	0.919		
Winter	1.243 (0.685–2.255)	0.474		
Pathogen				
InfA				
Negative	1 (reference)			
Positive	1.498 (0.548–4.099)	0.431		
InfB				
Negative	1 (reference)		1 (reference)	
Positive	3.641 (1.316–10.074)	0.013	3.009 (1.041–8.697)	0.042
HPIV				
Negative	1 (reference)		1 (reference)	
Positive	2.113 (1.127–3.962)	0.020	2.226 (1.170–4.235)	0.015
HBoV				
Negative	1 (reference)			
Positive	0.794 (0.110–5.739)	0.819		
HRV				
Negative	1 (reference)			
Positive	0.767 (0.472–1.245)	0.283		
ADV				
Negative	1 (reference)		1 (reference)	
Positive	1.978 (1.081–3.620)	0.027	2.035 (1.105–3.750)	0.023
HRSV				
Negative	1 (reference)			
Positive	1.614 (0.704–3.700)	0.258		
HMPV				
Negative	1 (reference)			
Positive	1.059 (0.259–4.324)	0.936		

Abbreviations: InfA, influenza A; InfB, influenza B; HPIV, human parainfluenza virus; HBoV, human bocaparvovirus; HRV, human rhinovirus; ADV, adenovirus; HRSV, human syncytial virus; HMPV, human metapneumovirus; OR, odds ratio; CI, confidence interval. Model 1 was not adjusted. Model 2 was adjusted for age, sex, period, season, and pathogens.

## Data Availability

The datasets generated during and/or analyzed during the current study are available from the corresponding author on reasonable request.

## Supplementary Materials

The following supporting information can be downloaded at: https://www.mdpi.com/article/10.3390/v17091266/s1, Figure S1: Screening and enrollment inpatients of flow chart; Figure S2: Epidemiological trends of nine respiratory pathogens from September 2021 to November 2024.

## Abbreviations

MP	mycoplasma pneumoniae
ARTIs	acute respiratory tract infections
SMPP	severe mycoplasma pneumoniae pneumonia
NPIs	non-pharmaceutical interventions
OR	odds ratio
CI	confidence interval
CAP	community-acquired pneumonia
SCH	Children’s Hospital of Soochow University
RT-PCR	real-time polymerase chain reaction
HPIV	human parainfluenza virus
HBoV	human bocaparvovirus
InfA	influenza A
InfB	influenza virus B
HRV	human rhinovirus
HRSV	human syncytial
HMPV	human metapneumovirus
ADV	adenovirus
