# Supplementary material for: Co-Detection of ADV, Influenza B, and HPIV: Independent Risk Factors for SMPP with Changes in NPIs
Source: Viruses. 2025 Sep 19;17(9):1266. doi: 10.3390/v17091266 (PMC12474053; doi:10.3390/v17091266)
Supplement: Supplementary file 1 [file viruses-17-01266-s001.zip › viruses-3823028-supplementary.pdf]

Supplemental Figures

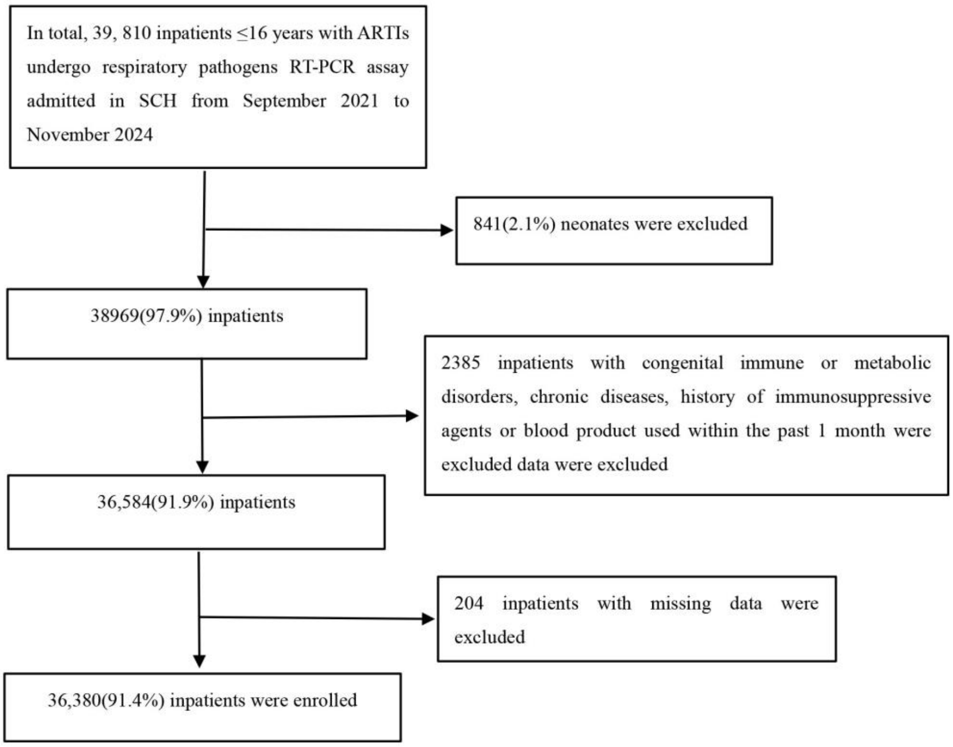

Fugure S1 Screening and enrollment inpatients of flow chart.

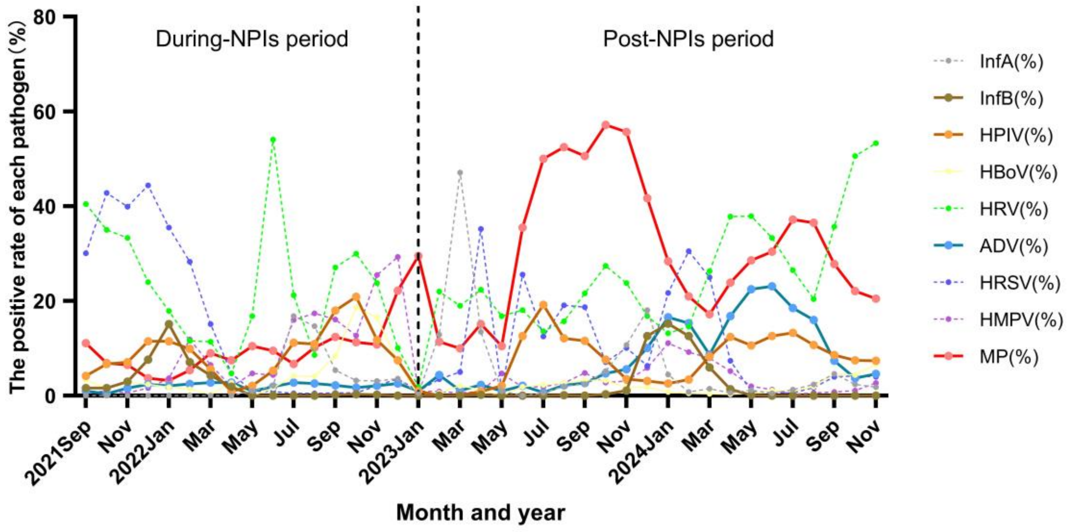

Figure S2 Epidemiological Trends of Nine Respiratory Pathogens from September 2021 to November 2024
